# Superior artificial synaptic properties applicable to neuromorphic computing system in HfO_*x*_-based resistive memory with high recognition rates

**DOI:** 10.1186/s11671-023-03862-0

**Published:** 2023-06-24

**Authors:** Hyun Kyu Seo, Su Yeon Lee, Min Kyu Yang

**Affiliations:** Artificial Intelligence Convergence Research Lab, Sahmyook University, 815 Hwarang-ro, Nowon-gu, Seoul, 01795 Republic of Korea

**Keywords:** ReRAM, Synaptic plasticity, Neuromorphic computing, Nonlinearity, Recognition rate

## Abstract

With the development of artificial intelligence and the importance of big data processing, research is actively underway to break away from data bottlenecks and modern Von Neumann architecture computing structures that consume considerable energy. Among these, hardware technology for neuromorphic computing is in the spotlight as a next-generation intelligent hardware system because it can efficiently process large amounts of data with low power consumption by simulating the brain’s calculation algorithm. In addition to memory devices with existing commercial structures, various next-generation memory devices, including memristors, have been studied to implement neuromorphic computing. In this study, we evaluated the synaptic characteristics of a resistive random access memory (ReRAM) with a Ru/HfO_*x*_/TiN structure. Under a series of presynaptic spikes, the device successfully exhibited remarkable long-term plasticity and excellent nonlinearity properties. This synaptic device has a high operating speed (20 ns, 50 ns), long data retention time (> 2 h @85 ℃) and high recognition rate (94.7%). Therefore, we propose that memory and learning capabilities can be used as promising HfO_*x*_-based memristors in next-generation artificial neuromorphic computing systems.

## Introduction

In the existing Von Neumann structure, information is exchanged through a data bus in the form of separate central processing units and memory. However, as the amount of data increases exponentially, memory bottlenecks become inevitable, preventing memory from maintaining the computational speed of the central processing unit [1, 2]. Therefore, the limitations of the von Neumann structure are revealed in algorithms that require the processing of vast amounts of data, such as artificial intelligence and deep learning [3]. Neuromorphic computing is a system that has been proposed to solve this problem. Neuromorphic computing mimics the biological functions of the human brain, and the implementation of synaptic devices and neuron circuits is essential [4–11]. Among these, synaptic devices have the most important analog characteristics that can express synaptic strength in various ways because of the number of signals or temporal correlation. The concept of a memristor was introduced to express these analog characteristics [12]. A memristor refers to an element whose resistance value changes according to an applied signal pulse and may also serve as a memory for storing the same. Memristor elements include ReRAM, magnetic random access memory (MRAM), and phase-change random access memory (PRAM), among which ReRAM has been actively studied as an artificial synapse device owing to its simple structure and high compatibility with complementary metal-oxide semiconductor (CMOS) [13–17]. HfO_2_ is a material with excellent reproducibility and repeatability and has long been studied in non-volatile storage memory applications as well as the high-k dielectric of CMOS [18–33]. An ideal synaptic memristor for neuromorphic computing should have high speed, endurance, and uniformity.

In this study, we propose a Ru/HfO_*x*_/TiN single layer ReRAM. Our proposed device showed nanosecond speed (20 ns, 50 ns) and stable retention characteristics (85 ℃, 2 h). We also showed ultra-low nonlinearity at the speed of nanoseconds and stable endurance that remains even after 50 k number of pulses. Based on these advantages, the recognition rate of 94.7% was confirmed in the MNIST-based object recognition simulation.

## Experimental

**Fig. 1:**
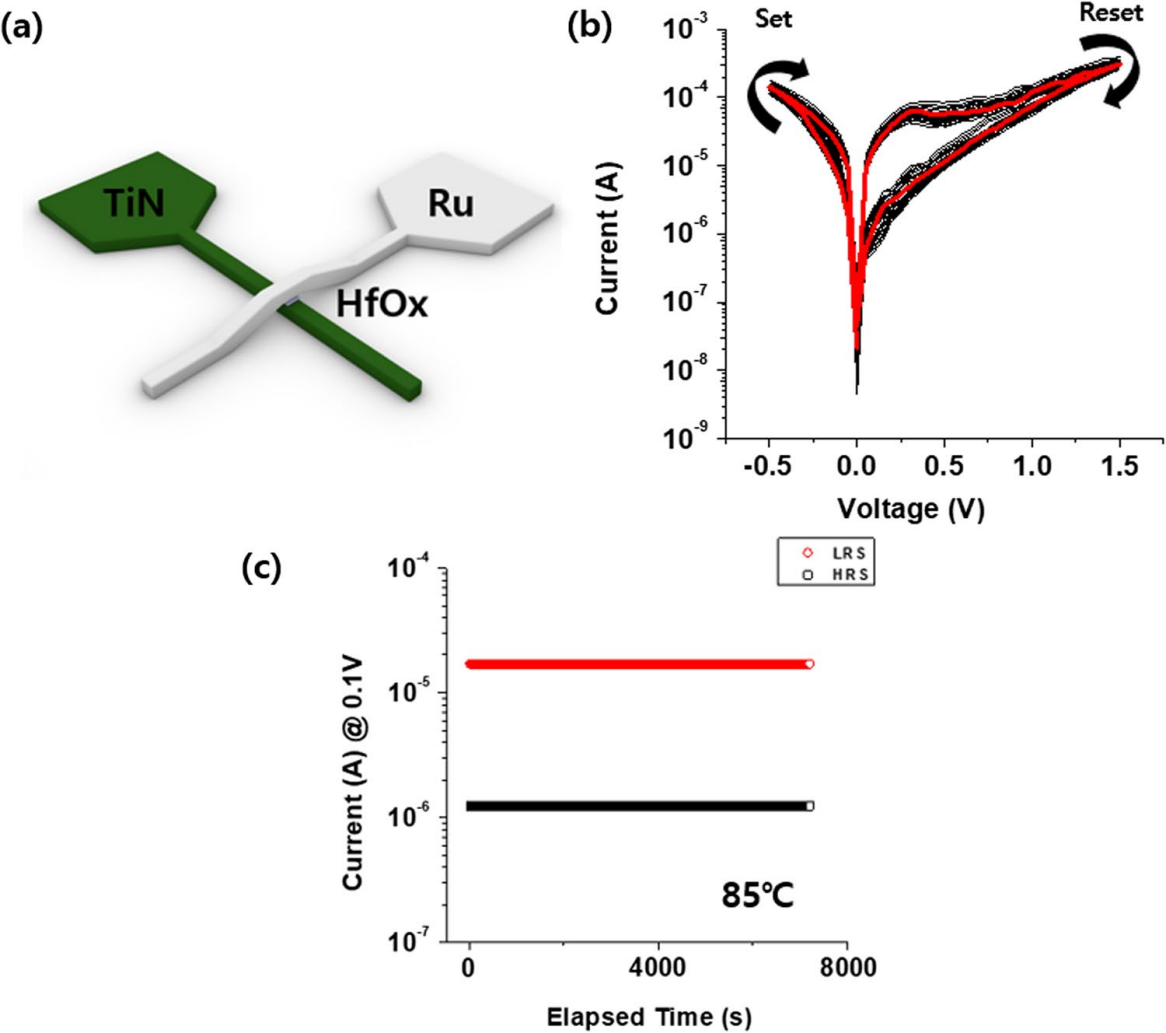
**a** Structure of the Ru/HfO_*x*_/TiN device. **b** I-V characteristics of the device by a negative set and positive reset. **c** Retention characteristics of the HRS and LRS

The fabricated resistive switching (RS) devices exhibited a crossbar-like structure with a 16 um^2^ cell size. To fabricate the RS device, a 150 nm-thick TiN layer was sputtered on a SiO_2_/Si substrate and patterned to a shape of crossbar-like structure type bottom electrode (BE). We patterned TiN BE using photolithography and then deposited the HfO_*x*_ layer using the physical vapor deposition (PVD) method. The 10 nm-thick HfO_*x*_ layer was formed using the radio-frequency (RF) reactive sputtering method. The base and working pressures for sputtering were maintained at 5e−7 and 1e−3 torr, respectively. The oxygen flow rate for HfO_*x*_ deposition was 6 sccm. After the HfO_*x*_ layer formation, the Ru top electrodes (TE) were patterned using the same process as that used for the BE, and the TE and BE were crossed perpendicularly to make the crossbar-type device. All electrical properties of our RS devices were tested using a semiconductor parameter analyzer (SPA, Keithley 4200 SCS) and an arbitrary function generator (AFG, Agilent 81150A). The RF circuit-switching module alternately accesses the two-terminal electric circuits between the SPA and AFG. All measurements were performed at room temperature under an ambient atmosphere. A bias was applied to the top Ru electrode, whereas the bottom TiN electrode was grounded. The Ru/HfO_*x*_/TiN structure is shown in Fig. 1a.

## Results and discussion

**Fig. 2:**
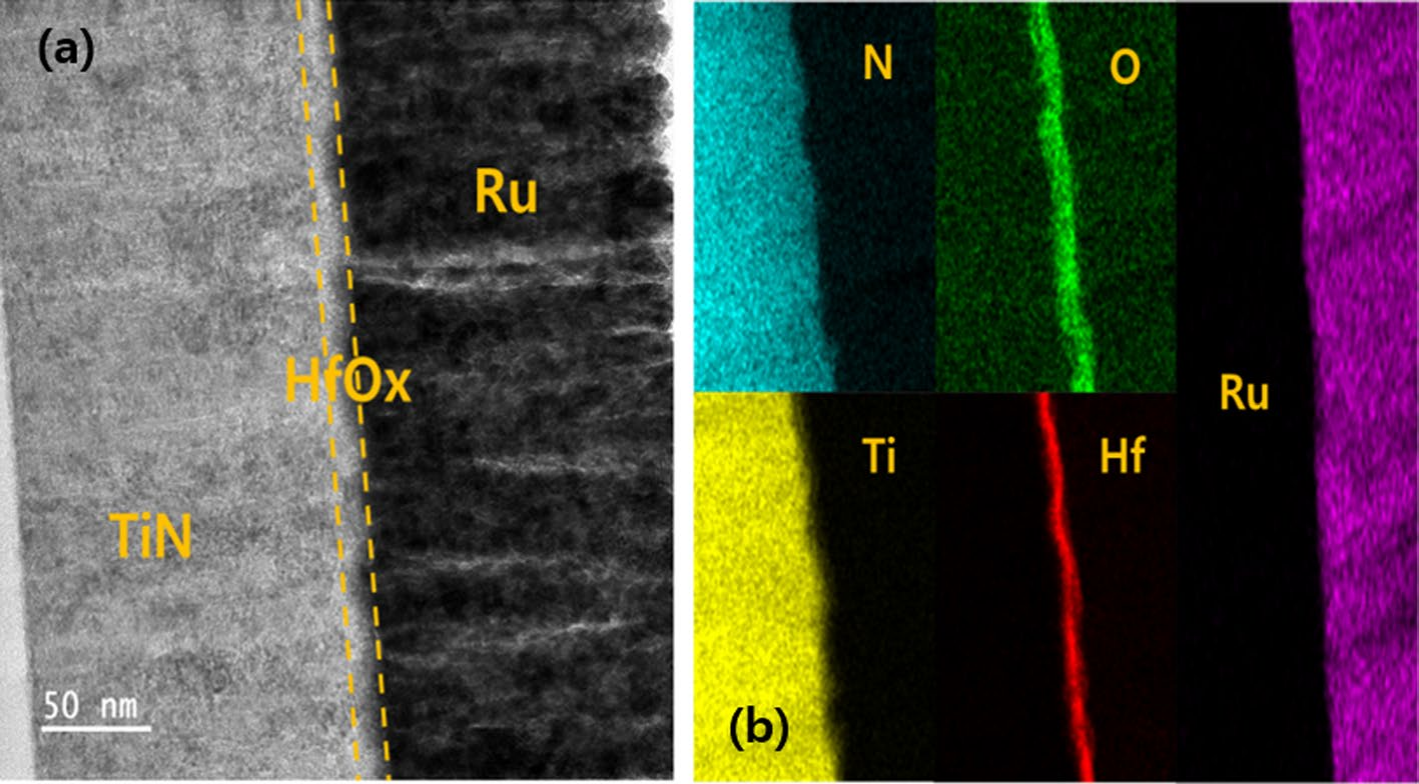
**a** Cross-sectional high-resolution transmission electron microscopy (HR-TEM) image of Ru/HfO_*x*_/TiN. **b** EDS elemental mapping

DC bias sweep of 100 cycles were applied to the device to characterize the typical I-V curves for electrical analysis, as shown in Fig. 1b. It shows a negative set and positive reset during − 0.5 V ~ 1.5 V. DC bias sweeps were applied to the device after the forming process. As shown in Fig. 1c, the retention characteristics of the HRS and LRS states were evaluated to confirm the storage maintenance characteristics of the device at 85℃. Several tests, such as long-term potentiation (LTP) and long-term depression (LTD), have been conducted on Ru/HfO_*x*_/TiN devices to mimic the synaptic functions essential for artificial electronic devices. The microscopic structure of the Ru/HfO_*x*_/TiN film was characterized using high-resolution transmission electron microscopy (HR-TEM), as shown in Fig. 2a. Figure 2b shows the distributions of the components (Ru, Hf, O, Ti and N) obtained through energy-dispersive X-ray spectroscopy (EDS) mapping.

The ReRAM is largely divided into filamentary and interface types. The filamentary type has advantages, such as low energy and fast switching on the nanosecond scale during programming [34–42]. In general, it rapidly changes to a low-resistance state when the filament is formed (set) and gradually changes to a high-resistance state when the filament is broken (reset). Analog switching characteristics must be used in a device; therefore, research is actively underway to use only the reset process, which causes gradual resistance changes, or to gradually change the rapid set process. The filamentary-type ReRAM is divided into oxide-based resistive memory (OxRAM), which is switched owing to defects in the oxide film, and conductive bridge random access memory (CBRAM), which is switched by metal ions. Unlike the filamentary-type ReRAM, the resistance of the interface-type ReRAM changes owing to the movement of oxygen ions at the interface between the metal and the insulator layer rather than the formation of a local channel in principle [43–48]. Therefore, because uniform switching characteristics are observed and the conductivity changes according to the amount of ion movement, it may have multiple resistance states. It also has the advantage of being able to secure the characteristics of gradually changing conductivity according to pulses. Our proposed device is an interface-type ReRAM caused by the movement of oxygen ions at the interface between the metal and insulator and shows gradual behavior in both set and reset operations. In addition, it differs from other interface types ReRAM in that it shows nanosecond-scale switching, which is an advantage of the filamentary type.

When potentiation and depression operations are performed, the linearity characteristics deteriorate if the conductivity of the device changes rapidly. In this case, the uniformity of the conductivity decreases and a negative effect on the accuracy of the pattern recognition rate becomes inevitable. The device may break down when abrupt resistance changes occur during the reset operations. To prevent this, it is common to set current compliance (C.C). However, in a pulse operation, it is impossible to set the current compliance itself; therefore, other methods, such as attaching an external load resistor, are required. In contrast, our device has the advantage of self-compliance without current compliance or an external load resistor. This self-compliance was due to the formation of oxygen reservoir at the interface between HfO_*x*_ and TiN [49, 50]. TiON produced by oxidation at the interface serves as an oxygen reservoir. In addition, owing to the gradual set and reset operations, a relatively constant change in conductance can be expected during the LTP and LTD operations.

**Fig. 3:**
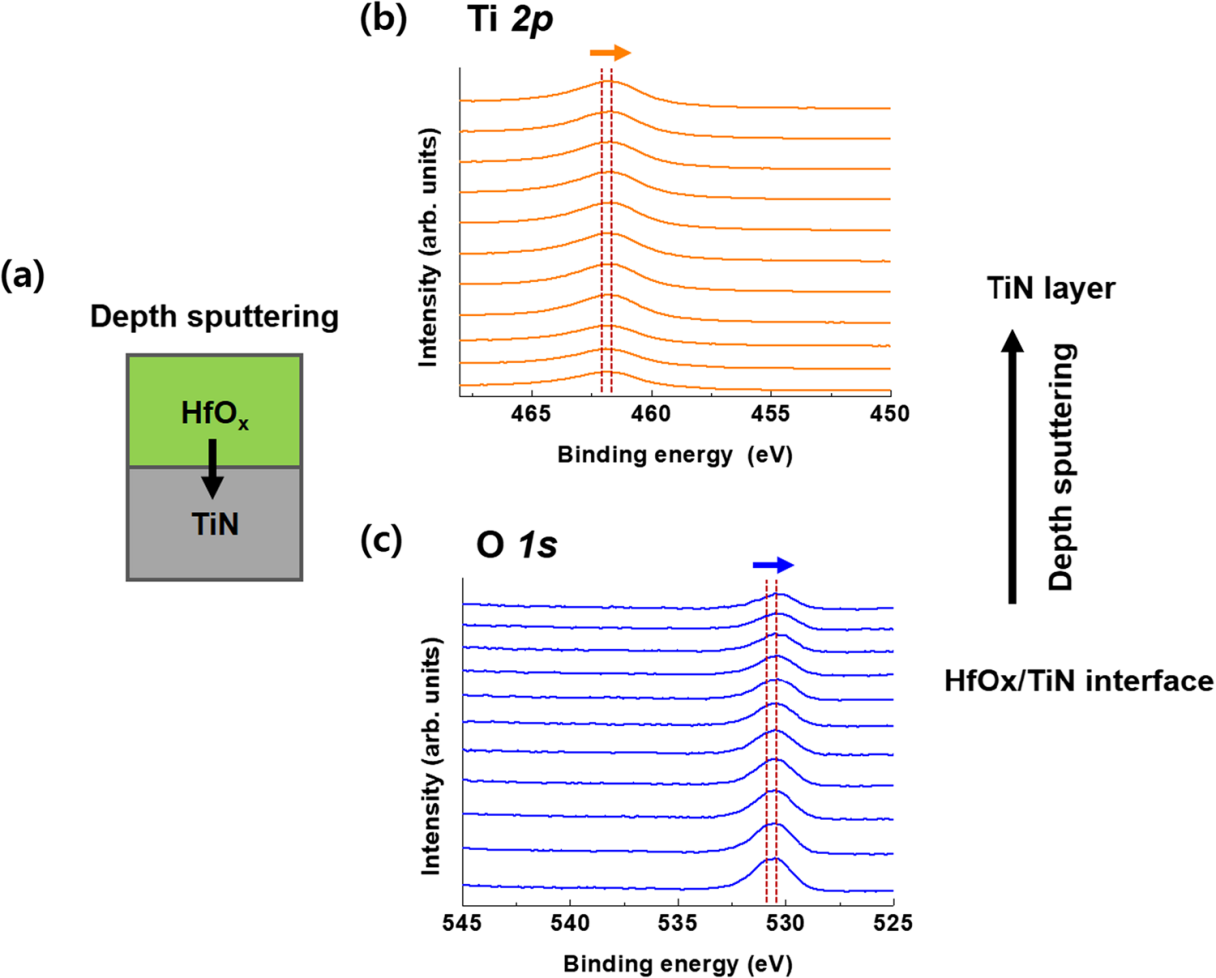
X-ray Photoelectron Spectrometer depth profile analysis at the interface of HfO_*x*_ and TiN. **a** Ti 2p and **b** O 1s peak

X-ray photoelectron spectrometer depth profile (XPS) analysis of identical material stacks with HfO_*x*_/TiN devices was performed to determine the microscopic role of the TiON layer in the device. Figure 3a shows a schematic of the sample structure used for XPS analysis. Figure 3b and c shows the variations in the in-depth XPS spectra of Ti 2p and O 1s, respectively. The direction of sputtering is denoted by an arrow on the right side of Fig. 3. As a result, the binding energy of Ti 2p decreased from 461.9 to 461.4 eV, and the binding energy of O 1s decreased from 530.6 to 530.1 eV. This analysis indicates that TiON was formed between the HfO_*x*_ and TiN layers. The set behavior occurring in the negative region was also formed by this TiON layer, which can perform a gradual set operation by acting as an oxygen reservoir [51, 52].

**Fig. 4:**
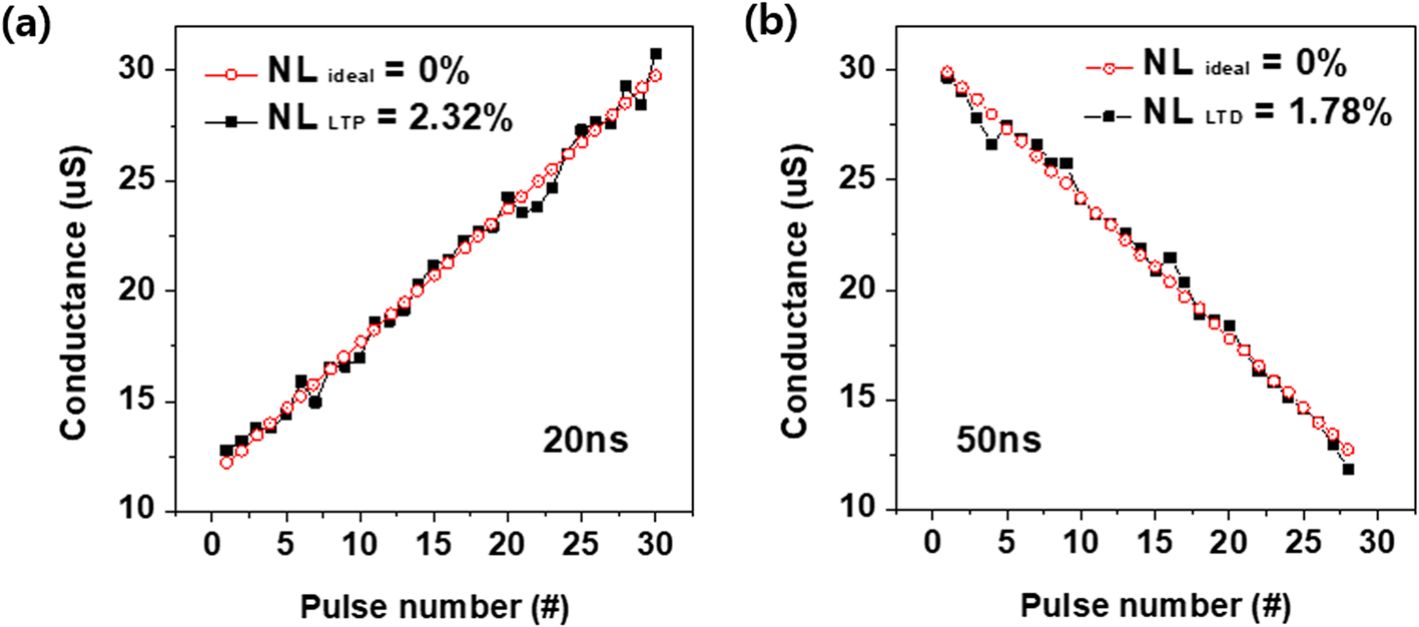
Conductance level of the Ru/HfO_*x*_/TiN device to extract nonlinearity **a** LTP and **b** LTD

Linear weight update for synaptic devices is important for achieving high learning accuracy in hardware neuromorphic systems. To evaluate the conductance updates in the LTP and LTD curves, nonlinearity (NL) was defined using the following equation:

$$\mathrm{NL}=\mathrm{average}\left(\left|\frac{\mathrm{G}-{G}_{\mathrm{Linear}}}{G}\right|\times 100 \%\right)$$

where *G* is the measured conductance value and GLinear is the conductance with an ideal weight update. The NL value of LTP was 2.32% and the LTD was 1.78%. We used the incremental pulse programming (ISPP) method to assess synaptic plasticity. ISPP refers to the process of increasing or decreasing the amplitude at a constant level at the same pulse width. As shown in Fig. 4a, 30 pulses were applied during the LTP operation. The pulse width was 20 ns, and the amplitude range was approximately − 1.45 to − 1.73 V. For the LTD operation, 28 pulses were applied, as shown in Fig. 4b. The pulse width was 50 ns, and the amplitude range was approximately 1.5–2.06 V.

**Fig. 5:**
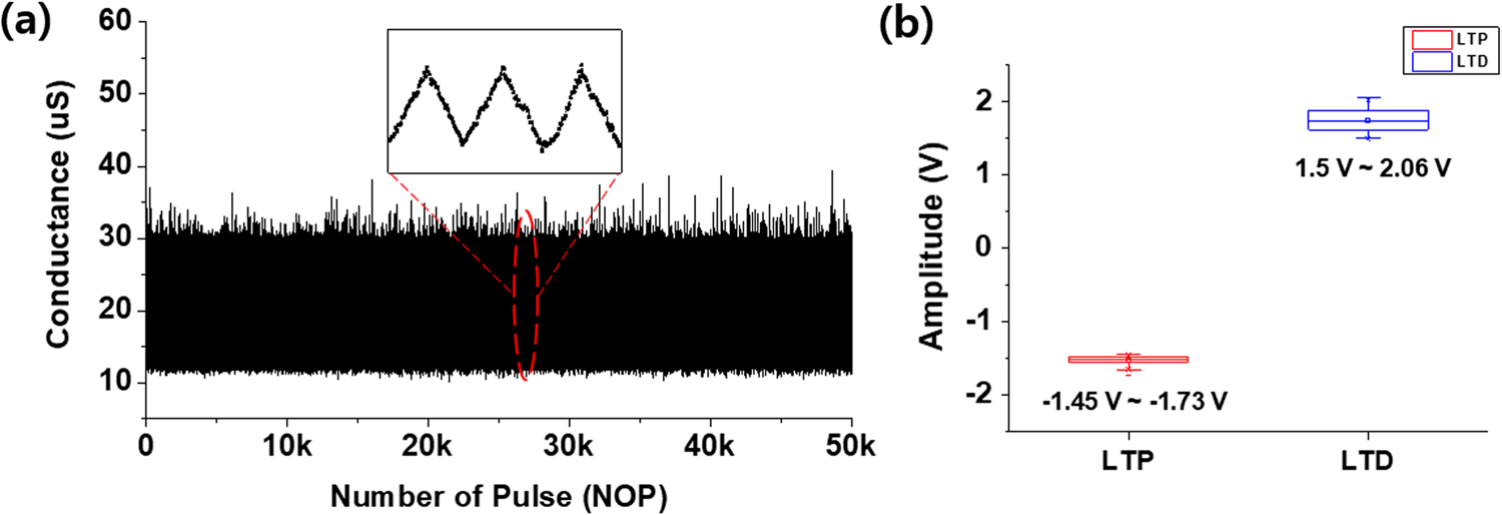
**a** The repetitive synaptic conductance variation characteristic of Ru/HfO_*x*_/TiN, inset image is LTP/LTD operation of the Ru/HfO_*x*_/TiN device. **b** Distribution of LTP/LTD amplitude authorized to ISPP

The synapse, acting as a neuronal junction or bridge, controls the electrical or chemical signals between two neurons. Synapses regulate the connection strength and are involved in learning and memory in the human brain. Similarly, a metal–insulator–metal (MIM) structure can facilitate weight (conductance) adjustment through external pulse inputs. LTP and LTD are key features in adjusting neuronal synaptic plasticity. Figure 5a shows the 50 k pulse number endurance, which indicates stable operation. The inset image shows the 3-cycle LTP and LTD curves driven by each pulse. The read voltage was measured at 0.1 V after applying the set pulse (− 1.45 to − 1.75 V, 20 ns). For LTD, a reset pulse (1.5–2.06 V, 50 ns) was applied, and a read voltage of 0.1 V was used. The conductance values were tuned in 12 µS of 30 µS. As shown in Fig. 5b, it can be observed that there is little difference in amplitude value even in several LTP and LTD operations, which shows that it always has a similar conductance value and that data can be safely stored.

**Fig. 6:**
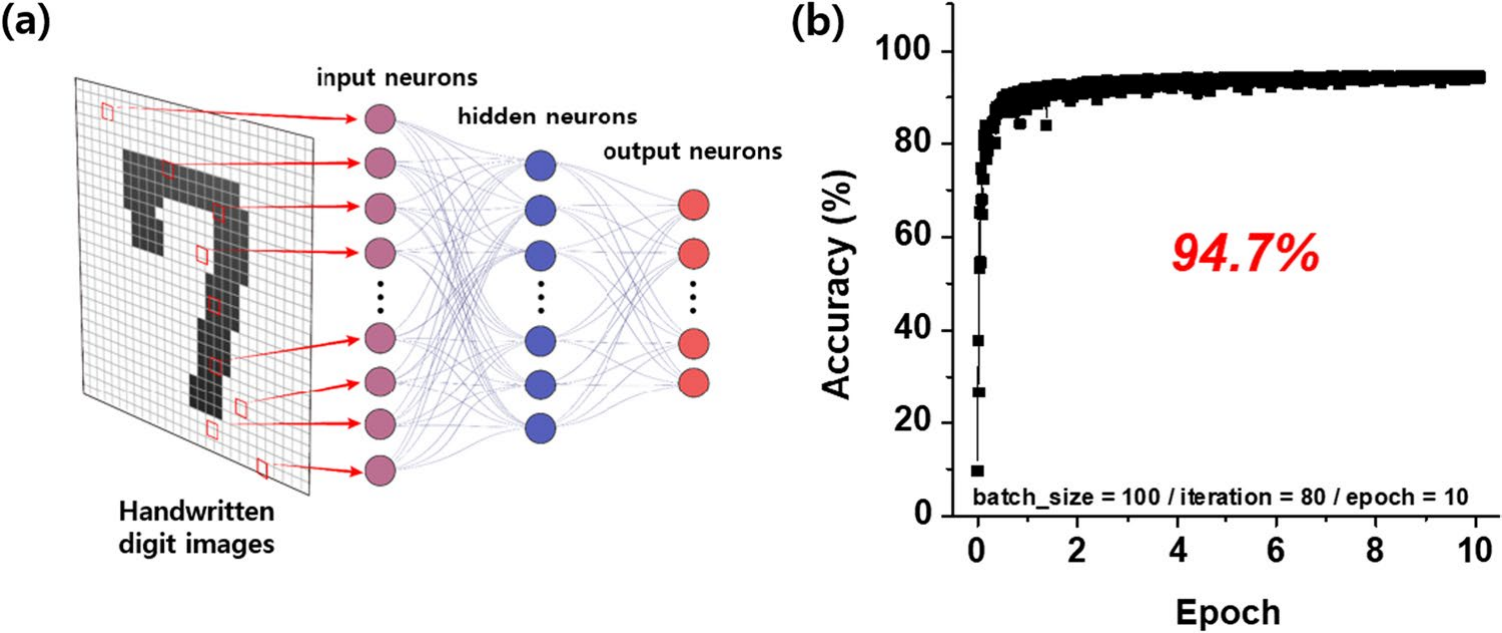
**a** Schematic of the structural concept of the inference simulation. **b** The inference simulation result of the Ru/HfO_*x*_/TiN device

The artificial neural network (ANN) perceptron structure using HfO_*x*_ as synapse, which consists of 784 input neurons, 200 hidden neurons and 10 output neurons. The ANN structure of a typical perceptron consists of three layers (input, hidden, and output layers), as shown in Fig. 6a. The input layer receives a value and does not specifically calculate. Therefore, if the model is simply composed of an input layer and an output layer, its accuracy will not be highly evaluated. The accuracy was increased by adding a hidden layer to the artificial neural network. In this ANN architecture, the modified national institute of standards and technology (MNIST) dataset was used to learn handwritten digit classification. In the MNIST dataset, handwritten digit images are represented by grayscale 28 × 28 pixel arrays. In the ANN learning process, the LTP and LTD characteristics of the device were used as synapses for handwriting recognition. The classification accuracy was then evaluated using a test dataset at each epoch. We divided 8000 pieces of data into 100 pieces each, 80 iteration is required for 1 epoch, and 10 parameter updates occur. The resulting accuracy was 94.7%, as shown in Fig. 6b, indicating that the device successfully operated as a synaptic device, representing the synaptic weights required for neural networks.

## Conclusion

In this study, we successfully fabricated an HfO_*x*_ film-based ReRAM device using RF magnetron sputtering with an HfO_2_ target. Long-term plasticity was simulated through electrical synapses, including LTP and LTD, by applying continuous pulses to the top electrodes. The synaptic operation of 50 k was stably performed at a high speed of 20 ns and 50 ns during the long-term potentiation and depression operations, respectively. Based on this, a high recognition rate of 94.7% was achieved through the LTP or LTD characteristics with ultralow nonlinearity. Therefore, we present the possibility of using HfO_*x*_-based resistive memory devices in neuromorphic computing, particularly for tasks that require real-time processing and low power consumption.

## Data Availability

The datasets generated during and/or analysed during the current study are available from the corresponding author on reasonable request.

## References

[1] Hasler J, Marr B (2013). Finding a roadmap to achieve large neuromorphic hardware systems. Front Neurosci.

[2] Jeong D, Kim K, Kim S, Choi B, Hwang C (2016). Memristor for energy-efficient new computing paradigms. Adv Electron Mater.

[3] Tang T, Xia L, Li B, Luo R, Chen Y, Wang Y, Yang H. Spiking neural network with RRAM: can we use it for real-world application?. In: 2015 Design, automation & test in europe conference & exhibition (DATE), 2015. Pp. 860–865.

[4] Roy K, Jaiswal A, Panda P (2019). Towards spike-based machine intelligence with neuromorphic computing. Nature.

[5] Yu S, Wu Y, Jeyasingh R, Kuzum D, Philip Wong H-S (2011). An electronic synapse device based on metal oxide resistive switching memory for neuromorphic computation. IEEE Trans Electron Devices.

[6] Kim M, Lee J (2018). Short-term plasticity and long-term potentiation in artificial biosynapses with diffusive dynamics. ACS Nano.

[7] Pei J, Deng L, Song S, Zhao M, Zhang Y, Wu S, Wang G, Zou Z, Wu Z, He W, Chen F, Deng N, Wu S, Wu Y, Yang Z, Ma C, Li G, Han W, Li H, Wu H, Zhao R, Xie Y, Shi L (2019). Towards artificial general intelligence with hybrid Tianjic chip architecture. Nature.

[8] Mead C (1990). Neuromorphic electronic systems. Proc IEEE.

[9] Davies M, Srinivasa N, Lin T, Chinya G, Cao Y, Choday SH, Dimou G, Joshi P, Imam N, Jain S, Liao Y, Lin C, Lines A, Liu R, Mathaikutty D, McCoy S, Paul A, Tse J, Venkataramanan G, Weng Y, Wile A, Yang Y, Wang H (2018). Loihi: a neuromorphic manycore processor with on-chip learning. IEEE Micro.

[10] Esser SK, Merolla PA, Arthur JV, Cassidy AS, Appuswamy R, Andreopoulos A, Berg DJ, Mckinstry JL, Melano T, Barch DR, Nolfo CD, Datta P, Amir A, Taba B, Flickner MD, Modha DS (2016). Convolutional networks for fast, energy-efficient neuromorphic computing. Proc Natl Acad Sci U S A.

[11] Furber J (2016). Large-scale neuromorphic computing systems. J Neural Eng.

[12] Indiveri G (2011). Neuromorphic silicon neurons and large-scale neural networks: challenges and opportunities. Front Neurosci.

[13] Mandal S, Saha A. Memristors act as synapses in neuromorphic architectures. In: 2016 international conference on communication and electronics systems (ICCES), 2016. pp. 1–5.

[14] Hu S, Wu S, Jia W, Yu Q, Deng L, Fu Y, Liu Y, Chen T (2014). Review of nanostructured resistive switching memristor and its applications. Nanosci Nano Technol Lett.

[15] Philip Wong H-S, Lee H, Yu S, Chen Y, Wu Y, Chen P, Lee B, Chen F, Tsai M (2012). Metal-oxide RRAM. Proc IEEE.

[16] Zhang W, Gao B, Tang J, Li X, Wu W, Qian H, Wu H (2019). Analog-type resistive switching devices for neuromorphic computing. Phys Status Solidi R.

[17] Moon K, Lim S, Park J, Sung C, Oh S, Woo J, Lee J, Hwang H (2019). RRAM-based synapse devices for neuromorphic systems. Faraday Discuss.

[18] Wedig A, Luebben M, Cho D, Moors M, Skaja K, Rana V, Hasegawa T, Adepalli K, Yildiz B, Waser R, Valov I (2016). Nanoscale cation motion in TaO_*x*_, HfO_*x*_ and TiO_*x*_ memristive systems. Nat Nanotechnol.

[19] Goux L, Kar G, Fantini A, Cagliani J, Blaise P, Belmonte A, Detavernier C, Degraeve R, Groeseneken G, Jurczak M (2011). Electronic switching in phase-change memories. Adv Mater.

[20] Chen Y, Lu W, Williams RS (2011). High-performance HfO_*x*_-based resistive switching memory for non-volatile memory applications. ECS Trans.

[21] Jo SH, Chang T, Ebong I, Bhadviya BB, Mazumder P, Lu W (2010). Nanoscale memristor device as synapse in neuromorphic systems. Nano Lett.

[22] Li G, Li Y, Gao X, Wang X, Liu M, Liu X (2016). Effect of HfO_*x*_ thickness on the performance of HfO_*x*_-based resistive random access memory. Microelectron J.

[23] Wang H, Wang X, Yang JJ, Wu Q, Lee MJ, Lu WD (2012). Electron/hole polarity control of resistive memory switching in vertical HfO_*x*_ nanoscale switch. ACS Nano.

[24] Yu S, Wu Y, Wang XJJ, Wong H-SP (2011). Compact modeling of conductive-bridge random access memory (CBRAM) for circuit design. IEEE Trans Electron Devices.

[25] Kim S, Lee B, Lee J, Lee H, Park J, Hwang H (2019). Comprehensive understanding of the switching mechanism in HfO_*x*_-based RRAM devices. Nanotechnology.

[26] Waser R, Aono M (2007). Nanoionics-based resistive switching memories. Nat Mater.

[27] Chen Y, Gao B, Shang J, Wang X (2019). High performance of HfO_*x*_-based RRAM devices with insertion of Al_2_O_3_ thin layer. J Mater Sci Mater Electron.

[28] Lee MJ, Lee CB, Lee D, Lee SR, Chang M, Hur JH, Kim YB, Kim CJ, Seo DH, Seo S, Chung UI, Yoo IK, Park BG (2011). A fast, high-endurance and scalable non-volatile memory device made from asymmetric Ta_2_O_5__−__*x*_/TaO_2__−__*x*_ bilayer structures. Nat Mater.

[29] Yu S (2013). HfO_*x*_-based resistive switching memory: a review. J Semicond.

[30] Nishi Y (2013). HfO_*x*_-based resistive memory. Jpn J Appl Phys.

[31] Lam CH (2016). A review on recent developments of hafnium oxide-based resistive switching memory. J Mater Sci Mater Electron.

[32] Park S (2017). Recent advances in HfO_*x*_-based resistive switching memory. J Mater Chem C.

[33] Liu Y (2017). Review on resistive switching in high-k dielectrics: Materials, mechanisms and performance. Mater Sci Eng R.

[34] Zhou YX, Li Y, Su YT, Wang ZR, Shih LY, Chang TC, Chang KC, Long SB, Sze SM, Miao XS (2017). Nonvolatile reconfigurable sequential logic in a HfO_2_ resistive random access memory array. Nanoscale.

[35] Lee HY, Lee SY, Lee S, Lee S, Kim S, Lee D, Cho DH, Im S (2009). Filamentary resistive switching in ultra-thin amorphous carbon film. Appl Phys Lett.

[36] Lin CH, Tsai MJ, Tsai MJ, Liao MJ, Wu YH, Chueh YL, King YC (2014). Filamentary and interface-type resistive switching in TiO_2_-based resistive switching devices. Nanotechnology.

[37] Zhang S, Chen S, Huang Y, Chen H (2011). Filamentary resistive switching behavior in an interface-type ZnO resistive memory. J Appl Phys.

[38] Ebadati T, Rahmanian RH, Liu Y, Xia Z, Yang JJ, Xia Q (2021). Memristive synapses for efficient spiking neural networks: filamentary versus homogeneous resistive switching. IEEE Trans Electron Devices.

[39] Gao F, Wu Y, Liu X, Zhang Z, Li L, Huang C, Tan C, Chen Y, Song L (2021). Electroforming-free, low-power and high-reliability filamentary-based RRAM with improved endurance and retention. Microelectron Eng.

[40] Ullah AR, Uddin MN, Alim A, Hong SH, Kim SJ (2021). Enhancement of bipolar filamentary resistive switching in Al/AlO_*x*_/ITO RRAM devices by tuning the interface quality. J Mater Sci Mater Electron.

[41] Zhang Z, Luo Y, Liu S, Zhang X, Lu W (2021). Non-volatile resistive switching with filamentary mechanism in 3D-printed metal-organic framework devices. J Mater Chem C.

[42] Lu M, Huang Y, Zhang X, Wang Y, Chen H (2021). Filamentary resistive switching in nanocrystalline TiO_2_ films for resistive random access memory. J Mater Sci Mater Electron.

[43] Yao J, Liu H, Zhang J, Guo H, Zhu X, Hu C, Huang R (2017). Interface-type resistive switching memories. Adv Mater.

[44] Jeong J, Lee SH, Seo DH, Lee SY, Kim M, Kim KM, Jeon JH, Lee HJ, Park SH (2011). Interface-type resistive switching memory with nanoscale conductive filament. Adv Mater.

[45] Han T, Liu Y, Xie L, Sun H, Wang X, Wang X, Liu M (2021). Intrinsic physical mechanism of interface-type resistive switching. Appl Phys Lett.

[46] Liu S, Liu H, Zhang Y, Shi S, Du L, Chen C, Chen Z, Zhang H (2021). Highly uniform and stable resistive switching in interface-type HfO_2_-based RRAM with novel electrode engineering. Appl Phys A.

[47] Kim W, Park S, Kwon Y, Lee H (2021). Engineering the TiO_2_/Pt interface for improving the performance of interface-type RRAM devices. J Electron Mater.

[48] Kim SS, Kim SJ, Seo DH, Lee JW, Park J, Jeong YH, Jang J, Lee HJ (2021). Enhanced memory performance of interface-type RRAM by surface modification of metal electrodes. IEEE Electron Device Lett.

[49] Zhao Z, Zhang Z, Wu X, Li L, Liu J, Li J, Lu Y (2021). Highly uniform and stable interface-type resistive switching in HfO_2_ based RRAM with NiO embedded layer. Appl Phys Lett.

[50] Sun C, Lu SM, Jin F, Mo WQ, Song JL, Dong KF (2018). Control the switching mode of Pt/HfO_2_/TiN RRAM devices by tuning the crystalline state of TiN electrode. J Alloy Compd.

[51] Kim S, Kim T-H, Kim H, Park B-G (2020). Current suppressed self-compliance characteristics of oxygen rich TiO_*y*_ inserted Al_2_O_3_/TiO_*x*_ based RRAM. Appl Phys Lett.

[52] Shin HJ, Seo HK, Lee SY, Park M, Park S-G, Yang MK (2022). Quad-level cell switching with excellent reliability in TiN/AlO_*x*_:Ti/TaO_*x*_/TiN memory device. Materials.

